# Analysis of Case-Parent Trios Using a Loglinear Model with Adjustment for Transmission Ratio Distortion

**DOI:** 10.3389/fgene.2016.00155

**Published:** 2016-08-31

**Authors:** Lam O. Huang, Claire Infante-Rivard, Aurélie Labbe

**Affiliations:** ^1^Department of Epidemiology, Biostatistics and Occupational Health, McGill UniversityMontréal, QC, Canada; ^2^Department of Psychiatry, McGill UniversityMontréal, QC, Canada; ^3^Douglas Mental Health University InstituteMontréal, QC, Canada

**Keywords:** Transmission Ratio Distortion, meiotic drive, family-based association analysis, log-linear model, case-parent triad, case-parent trios, intrauterine growth restriction, intrauterine growth retardation

## Abstract

Transmission of the two parental alleles to offspring deviating from the Mendelian ratio is termed Transmission Ratio Distortion (TRD), occurs throughout gametic and embryonic development. TRD has been well-studied in animals, but remains largely unknown in humans. The Transmission Disequilibrium Test (TDT) was first proposed to test for association and linkage in case-trios (affected offspring and parents); adjusting for TRD using control-trios was recommended. However, the TDT does not provide risk parameter estimates for different genetic models. A loglinear model was later proposed to provide child and maternal relative risk (RR) estimates of disease, assuming Mendelian transmission. Results from our simulation study showed that case-trios RR estimates using this model are biased in the presence of TRD; power and Type 1 error are compromised. We propose an extended loglinear model adjusting for TRD. Under this extended model, RR estimates, power and Type 1 error are correctly restored. We applied this model to an intrauterine growth restriction dataset, and showed consistent results with a previous approach that adjusted for TRD using control-trios. Our findings suggested the need to adjust for TRD in avoiding spurious results. Documenting TRD in the population is therefore essential for the correct interpretation of genetic association studies.

## Introduction

Transmission Ratio Distortion (TRD) occurs when the transmission of alleles from a heterozygous parent to the offspring statistically deviates from the Mendelian Law of Inheritance. TRD results from disruptive mechanisms occurring during gametic and embryonic development (Huang et al., 2013), including germline selection (Hastings, 1991), meiotic drive (Pardo-Manuel de Villena and Sapienza, 2001), gametic competition (Zöllner et al., 2004), embryo lethality (Zöllner et al., 2004), and imprint resetting error (Naumova et al., 2001; Yang et al., 2008). The presence of TRD leads to spurious conclusions in association studies.

A recent study uses a Bayesian framework to model TRD in boars and piglets and was shown to achieve appealing statistical performance (Casellas et al., 2014). In humans, individuals unselected for phenotype have been studied to detect TRD in the general population, such as in the Framingham Heart study (Paterson et al., 2009; Meyer et al., 2012), the Centre d'Etude du Polymorphisme Humain (Naumova et al., 2001; Yang et al., 2008), the HapMap project (The International HapMap Consortium, 2005), and the 1000 Genomes Project (Auton et al., 2005).

In family-based study design, Transmission Disequilibrium test (TDT; Spielman et al., 1993) is among the most well-known linkage disequilibrium tests. It is a McNemar test of transmitted vs. untransmitted alleles from parents to an affected child. It was originally developed to test both linkage and association at a marker locus by studying case-parent trios. The usage of TDT became wide-spread since its inception because of its simplicity and robustness to population stratification. There have been multiple extensions of TDT to address multi-allelic loci (Sham and Curtis, 1995; Wilson, 1997; Lazzeroni and Lange, 1998), multiple marker loci (Lazzeroni and Lange, 1998), quantitative traits (Allison, 1997; Rabinowitz, 1997; Xiong et al., 1998), nuclear family with multiple affected children (Martin et al., 1997) and unaffected siblings (Lazzeroni and Lange, 1998), pedigrees (Sham and Curtis, 1995), late-onset diseases (Spielman and Ewens, 1998), and imprinting effect (Hu et al., 2007).

In some studies, case and control populations were analyzed separately to detect a difference in transmission (Friedrichs et al., 2006; Shoubridge et al., 2012). To address the possible presence of TRD in the studied population, Spielman et al. (1993) analyzed both case/control-trios separately using the TDT. True association was then assessed using a Pearson's Chi-square test. Deng and Chen (2001) proposed a TDT statistic that is the sum of TDT statistics for case/control-trios for similar purpose. Previously, we also suggested a modified TDT statistics where the two diagonal counts in McNemar test are multiplied by *t* and (1−*t*), respectively, where *t* is the transmission ratio of the minor allele in control-trios (Labbe et al., 2013).

Other statistical measures have also been proposed to study affected offspring, such as Binomial exact test (Dean et al., 2006; Yang et al., 2008), Pearson's Chi-square test (Imboden et al., 2006; Bettencourt et al., 2008), multipoint non-parametric linkage (NPL) test (Paterson and Petronis, 1999; Paterson et al., 2003), Mann-Whitney *U*-test (De Rango et al., 2007), and multivariate logistic model (Yang et al., 2008). These methods only give statistical significance of linkage and association, but do not estimate the disease relative risk (RR). Relative risk is considered as an important information because it measures the difference in risk between individuals of different genotypes.

The family-based association test (FBAT; Lazzeroni and Lange, 1998; Rabinowitz and Laird, 2000) and likelihood methods that use case-trios to construct conditional logistic (Cordell et al., 2004), unconditional logistic (Weinberg, 1999), and loglinear models (Weinberg et al., 1998; Sinsheimer et al., 2003; Gjessing and Lie, 2006; Kistner et al., 2006, 2009) have also been used in family-based studies. In particular, Weinberg et al. proposed a loglinear model to detect an association between a marker and disease (Weinberg et al., 1998). This model estimates a RR of disease for the offspring, assuming Mendelian transmission. Unlike the other tests and models, it has a probability component that can be easily extended to adjust for TRD. Our proposed method uses the transmission ratio of a minor allele in control-trios, obtained from an external dataset such as HapMap (The International HapMap Consortium, 2005), 1000 Genomes Project phase 3 data (Auton et al., 2005), and family units in Framingham Heart Study (2008). These datasets are publically available and include healthy trios, which provide transmission ratio of alleles from parents to child, can be used to account for TRD through an offset in the model. There are others consortia with genome-wide data, but they are based mostly on unrelated individuals (Cavalli-Sforza, 2005; Prüfer et al., 2014), a few trios (Drmanac et al., 2010), large pedigrees (Drmanac et al., 2010; T2D-GENES Consortium TD-G, 2016) or diseased individuals (The Cancer Genome Atlas, 2016; T2D-GENES Consortium TD-G, 2016), which are neither adequate nor appropriate for our study on TRD.

This extended loglinear model was validated through extensive simulation studies and applied to an intrauterine growth restriction (IUGR) case-control study augmented with a case/control-trio study (Infante-Rivard et al., 2002; Infante-Rivard and Weinberg, 2005), investigating the role of thrombophilic genes in IUGR. The current literature in support of the association between thrombophilia and IUGR is inconsistent. We explored the possible role of TRD in these inconsistencies.

## Materials and methods

We investigated the association between a bi-allelic codominant disease susceptibility locus (DSL) and a disease, of which individuals express distinct disease risk associated with each of the three possible genotypes at the DSL. We defined genotype by the number of copies of the minor allele.

### Loglinear model by Weinberg et al. (1998)

The loglinear model proposed by Weinberg et al. (1998) assumes Mendelian transmission and mating symmetry, but not Hardy-Weinberg Equilibrium (HWE). We considered the simpler form of this model with only child genotype parameters.

In this model, the response variable is the number of trios for the 15 mother-father-child (*MFC*) genotype categories (Table 1). These 15 categories can be subdivided into six parental mating types. Covariates entering the model include two indicator variables for child genotypes 1 and 2, and five for mating types. The model which includes an intercept and an offset, is described as:

(1) $$\begin{array}{l} log\text{​}\left\{E\left[n_{MFC}|D\right]\right\}=\rho_{6}\;+\;\sum_{j\;=\;1}^{5}\rho_{j}I_{\left[S\;=\;j\right]}+log\left(2\right)I_{\left[MFC\;=\;111\right]}\\ \;+\;\beta_{1}I_{\left[C\;=\;1\right]}+\beta_{2}I_{\left[C\;=\;2\right]} \end{array}$$

*n*_*MFC*_ is the number of trios with genotypes *MFC*, and *D* is the disease status of the child. The ρ_*j*_ + ρ_6_ terms are the regression coefficients for the first five parental mating types; ρ_6_ is the intercept for the 6th mating type *MF* = 00; β_1_ and β_2_ are the regression coefficients for child genotypes 1 and 2, where β_1_ = *log (R*_**1**_*)* and β_2_ = *log (R*_**2**_*). R*_**1**_ and *R*_**2**_ are the RR with respect to genotype 0. This model 1, operates under the assumption of Mendelian transmission [derived in Appendix Derivation of Model 1 (Without TRD Offset) and 2 (With TRD Offset) and Table 6 in Supplementary Materials].

**Table 1:** **Relative risk, stratum frequency, and probability of transmission (TRD or Mendelian) for Case-parent trios study design**.

**Stratum**	***MFC* genotype**	**Stratum frequency under HWE**	**Probability of transmission (τ_*MFC*_) under TRD**	**Probability of transmission (τ_*MFC*_) under Mendelian**	**Relative risk**
1	222	*p*^4^	1	1	*R_2_*
2	212	2*p*^3^(1–*p*)	*t*	1/2	*R_2_*
	211		1–*t*	1/2	*R_1_*
	122		*t*	1/2	*R_2_*
	121		1–*t*	1/2	*R_1_*
3	201	*p*^2^(1–*p*)^2^	1	1	*R_1_*
	021		1	1	*R_1_*
4	112	4*p*^2^(1–*p*)^2^	*t*^2^	1/4	*R_2_*
	111		2*t*(1–*t*)	1/2	*R_1_*
	110		(1–*t*)^2^	1/4	1
5	101	2*p*(1–*p*)^3^	*t*	1/2	*R_1_*
	100		1–*t*	1/2	1
	011		*t*	1/2	*R_1_*
	010		1–*t*	1/2	1
6	000	(1–*p*)^4^	1	1	1

### Loglinear model with adjustment for TRD

Without the assumption of Mendelian transmission, model 1 can be generalized into:

(2) $$\begin{array}{l} log\text{​}\left\{E\left[n_{MFC}|D\right]\right\}=\xi_{6}+\sum_{j\;=\;1}^{5}\xi_{j}I_{\left[S\;=\;j\right]}+log\;\tau_{MFC}+\beta_{1}I_{\left[C\;=\;1\right]}\\ \;+\;\beta_{2}I_{\left[C\;=\;2\right]} \end{array}$$

where τ_*MFC*_ is the transmission offset *P*[*C*|*MF*], ξ_*j*_ + ξ_6_ terms (*j* = 1–5) are the regression coefficients for the first five mating types, and ξ_6_ is the intercept corresponding to the 6th mating type. The coefficients β_1_ and β_2_ are as defined in model 1. This model 2 accounts for TRD [derived in Appendix Derivation of Model 1 (Without TRD Offset) and 2 (With TRD Offset) and Table 6 in Supplementary Materials].

The offset τ_*MFC*_ depends on the TRD ratio *t*, defined as the transmission probability of a minor allele from a heterozygous parent to the child. This leads to a different offset in each *MFC* genotype category. The parameter *t* can take on values different from 0.5, and *t* = 0.5 corresponds to Mendelian transmission, in which case models 1 and 2 are equivalent [see Appendix Derivation of Model 1 (Without TRD Offset) and 2 (With TRD Offset) and Table 6 in Supplementary Materials].

We fitted both loglinear models (1) and (2) to obtain estimates *R*_**1**_ and *R*_**2**_, and their corresponding *Z*-test *p*-values. To assess significance of the association between the disease and the DSL, a Likelihood Ratio Test (LRT) was used [see Appendix Non-Central Chi-Square Likelihood for Model 1 (Without TRD Offset) and Model 2 (With TRD Offset) for the distribution of the LRT under the null and alternative hypotheses].

### Simulation study

A simulation study was set up for different TRD scenarios, where RR parameters, *p*-values, LRT *p*-values, Type 1 error, and power were compared between the 2 models, and the true *t* was used in model 2. A sensitivity analysis was also carried out to test the impact on RR estimates and power when an incorrect *t* is used.

#### Simulation setup

We considered a causal locus with no recombination. Disease prevalence is 0.1 for low penetrant common disease, and 0.01 for high penetrant rare disease. 100,000 trios were generated where 500 case-trios were sampled. Parental genotypes at the DSL were generated under HWE assuming a minor allele frequency (MAF) 0.1. The parameter *t* was specified between 0.1 and 0.9. Offspring were assigned to diseased or non-diseased phenotypes using risk associated with genotypes 0, 1, and 2, as *f*_**0**_, *f*_**1**_, and *f*_**2**_, respectively. The simulation was repeated 100 times and averaged RR estimates, *p*-value of the averaged *Z* statistics for RR and *p*-value of the averaged LRT statistics are reported.

#### Measuring impact of TRD on association statistics

We compared the RR, 95% CI, *p*-value and LRT *p*-value of both models under two scenarios: (1) a common disease associated of low penetrance at *f*_**0**_ = 0.1, *f*_**1**_ = 0.11, *f*_**2**_ = 0.15, and (2) a rare disease of high penetrance at *f*_**0**_ = 0.1, *f*_**1**_ = 0.5, *f*_**2**_ = 0.5. In scenario (2), a dominant model was assumed. To measure the inflation in RR and LRT *p*-values in model 1, we computed the log ratio of RR and LRT *p*-values in model 1 vs. 2. We also varied *f*_**1**_ fixing *f*_**2**_ = 0.15 to describe the corresponding inflation of LRT *p*-values. To assess the inflation of Type 1 error, we set the penetrance factors to *f*_**0**_ = *f*_**1**_ = *f*_**2**_ = 0.1 assuming no association while varying *t* from 0.1 to 0.9, using sample sizes of 100, 300, and 500. Finally, we evaluated the power of both models to detect a true association signal in the presence of TRD, by setting *f*_**0**_ = 0.1, *f*_**1**_ = 0.2, *f*_**2**_ = 0.3, varying *t* from 0.1 to 0.9 in the simulation. Critical value for declaring significance was α = 0.05.

#### Sensitivity analysis

The assumption in the simulation study was that true *t* is known. We examined the consequences of a misspecification of *t* on the RR estimates and the power, simulating three scenarios with true association signal, *f*_**0**_ = 0.1, *f*_**1**_ = 0.2, *f*_**2**_ = 0.3, and true *t* = 0.3, 0.5, or 0.7. For each scenario, model 2 was fitted with the offset τ_*MFC*_ calculated using a selected *t* varying between 0.1 and 0.9. We then evaluated the log ratio of RR and power obtained from model 2 using selected *t*-values vs. true *t* that adjust for TRD.

### Application of models 1 and 2 to a real dataset

We applied our model to the IUGR study described previously (Sapru et al., 2009; Kvasnicka et al., 2012). Cases were below 10th percentile according to weight whereas controls were selected at the same hospital and measured at or above the 10th percentile. DNA was obtained from parents of both cases and controls. The investigation pertained to the role of thrombophilic genes in IUGR. We examined six thrombophilic genes: Coagulation Factor XIII, A1 polypeptide (*F13A1*), Plasminogen activator inhibitor type 1 (*PAI-1*), Methylenetetrahydrofolate reductase variant A1298C (*MTHFR A1298C*), Methylenetetrahydrofolate reductase variant C677T (*MTHFR C677T*), Coagulation Factor V (*F5*), and Coagulation Factor II (*F2*). We computed the MAF using all complete trios and *t* using control-trios. We compared our extended model 2 with another method proposed by Infante-Rivard and Weinberg (2005) to quantify the extent of TRD in the same IUGR population, specifically for *F5*. The difference between our model 2 and the model used in Infante-Rivard and Weinberg (2005) is that the former inserts *t* as an offset in the loglinear model fitted with case-trios only, while the latter uses both case- and control-triosadding an interaction term between child genotype and case status.

This study was carried out in accordance with the recommendations of Le Comité d'éthique de la recherche, Centre Hospitalier Universitaire, Hôpital Sainte-Justine, Montréal, Québec, Canada. The protocol was approved by the same committee.

## Results

### Simulation study

#### Inflation of RR estimates and LRT *P*-values

**Figure 1:**
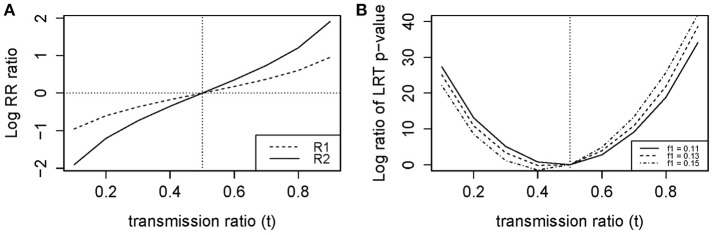
**Log ratio of (A) RR and (B) LRT *P*-values for models 1 (Unadjusted) vs. 2 (Adjusted)**.

When the transmission ratio was Mendelian, models 1 and 2 yielded the same RR and 95%CI (Tables 2, 3). When testing *t* = 0.3 where the disease allele is under-transmitted, the RR for model 1 was attenuated excluding 1 in the 95% CI, whereas RR estimates, *p*-values and LRT *p*-values were restored in model 2. Similarly, for *t* = 0.7, the RR for model 1 were inflated and this inflation was removed under model 2. The RR inflation ratio changes exponentially with respect to *t*, implying that even small deviation from *t* = 0.5 can lead to a substantial inflation (Figure 1A). The slope of RR ratio for *R*_**2**_ was double that of *R*_**1**_, showing that TRD affected *R*_**2**_ more severely than *R*_**1**_. In Figure 1B, when TRD is not adjusted for, the significance of the LRT *p*-values was inflated when *t* deviates from 0.5.

**Table 2:** **Relative risk with 95% CI, *P*-values, and likelihood ratio test *P*-values of models 1 (Unadjusted) and 2 (Adjusted) for a low penetrance common disease**.

***t***	**Model**	***R_1_***	**95% CI**	***P*-value**	***R_2_***	**95%CI**	***P*-value**	**LRT *P*-value**
0.3	1	0.47	0.33, 0.65	6.00E-06	0.25	0.06, 1.08	0.07	2.85E-06
	2	1.09	0.78, 1.51	0.59	1.34	0.30, 5.84	0.51	0.28
0.5	1	1.10	0.81, 1.51	0.53	1.40	0.51, 3.89	0.43	0.26
	2	1.10	0.81, 1.51	0.53	1.40	0.51, 3.89	0.43	0.26
0.7	1	2.52	1.78, 3.57	2.00E-07	8.01	3.18, 20.2	8.27E-06	6.57E-10
	2	1.08	0.76, 1.53	0.7	1.47	0.58, 3.70	0.42	0.25

R_1_, RR of cases carrying 1 copy of disease allele; R_2_, RR of cases carrying two copies of disease allele; Simulated with t = 0.3, 0.5, and 0.7 and population parameters: p = 0.1, f_0_ = 0.1, f_1_ = 0.11, f_2_ = 0.15.

**Table 3:** **Relative risk with 95% CI, *P*-values, and likelihood ratio test *P*-values of models 1 (Unadjusted) and 2 (Adjusted) for a high penetrance rare disease**.

***t***	**Model**	***R_1∕2_***	**95%CI**	***P*-value**	**LRT *P*-value**
0.3	1	2.44	1.20, 4.94	0.014	0.025
	2	5.71	2.82, 11.57	1.29E-06	8.62E-07
0.5	1	5.58	2.55, 12.21	1.55E-05	6.55E-07
	2	5.58	2.55, 12.21	1.55E-05	6.55E-07
0.7	1	13.73	4.99, 37.79	1.57E-07	2.62E-13
	2	5.87	2.13, 16.16	0.000504	2.23E-05

R_1/2_, RR of cases carrying one or two copies of disease allele; Simulated with t = 0.3, 0.5, and 0.7 and population parameters: p = 0.01, f_0_ = 0.1, f_1_ = 0.5, f_2_ = 0.5; Data is fitted with a dominant genotype model.

#### Inflation of type 1 error

**Figure 2:**
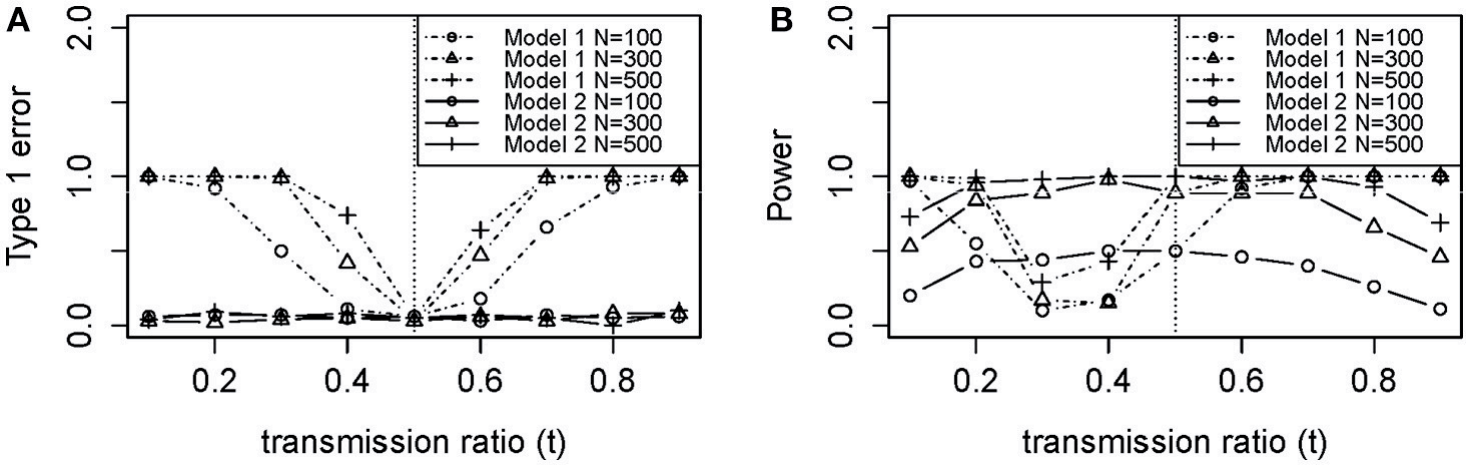
**Empirical (A) type 1 error and (B) power of models 1 (Unadjusted) and 2 (Adjusted)**.

**Figure 3:**
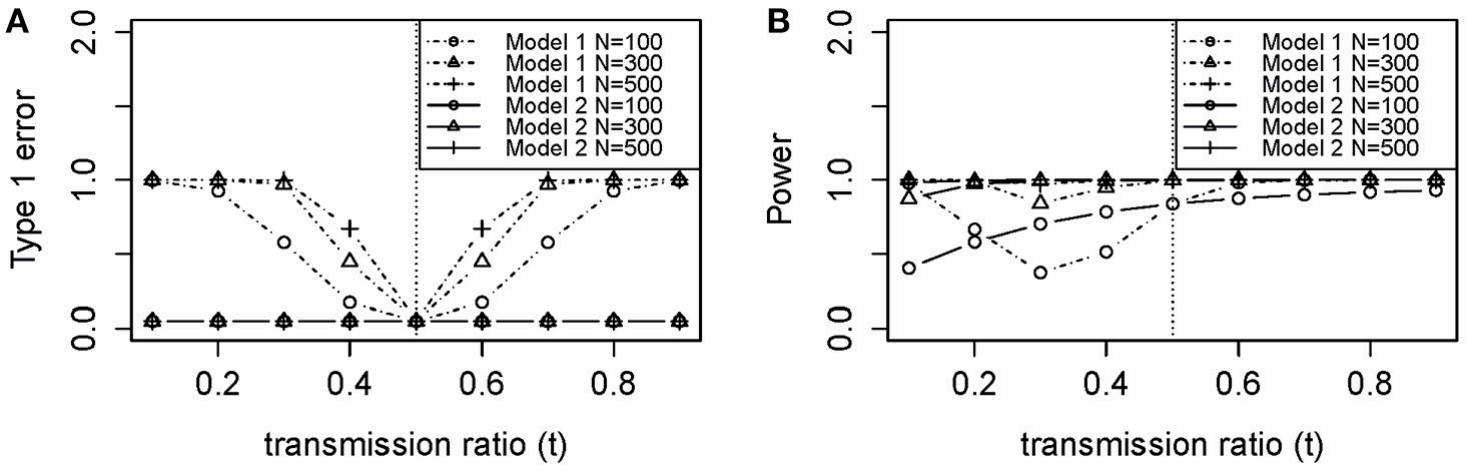
**Theoretical (A) type 1 error and (B) power of models 1 (Unadjusted) and 2 (Adjusted) using Equation (A6) and (A7) in Appendix**. **(A)** Type 1 Error (no association between disease and DSL where *f*_**0**_ = *f*_**1**_ = *f*_**2**_ = 0.1). **(B)** Power (true association between disease and DSL where *f*_**0**_ = 0.1, *f*_**1**_ = 0.2, *f*_**2**_ = 0.3). *N*, sample size (100, 300, and 500); *f*_**0**_, penetrance for genotype 0 individuals; *f*_**1**_, penetrance for genotype 1 individuals; *f*_**2**_, penetrance for genotype 2 individuals.

Figure 2A shows the empirical Type 1 Error we observed by fitting the loglinear model which is similar to our theoretical results in Figure 3A. Type 1 Error of the TRD-adjusted model 2 remained the same across all *t*-values, and were exactly the same for all sample sizes. Type 1 Error for model 2 does not depend on sample size or *t*, meaning that this model is robust to the effect of TRD when the null hypothesis is true. In Figure 2A, Type 1 Error for the unadjusted model 1 increased as *t* deviated from 0.5 which led to a false inflation of the association signals.

#### Power loss

Power for sample size *n* = 100 was poor in Figure 2B, with or without TRD. We also noticed that model 2 gave relatively stable power in the range of *t*, while model 1 power suffered from the effect of TRD. However, when *t* was lower than 0.2 or >0.5, model 1 power was greater than that of model 2. This is because a strong TRD actually inflates the power of detecting an association signal in either direction. Power for model 2 decreased slightly when *t* > 0.7, which suggested that the TRD offset overcompensates the inflation in power. However, a TRD ratio as large as 0.9 is rare, but even when *t* = 0.8, the power was still maintained around 0.8 for sample sizes of 300 and 500. Therefore, the power for model 2 was still adequate for a *t* between 0.2 and 0.8. Relatively consistent results were obtained between theoretical power (Figure 3B) and empirical power (Figure 2B).

#### Sensitivity analysis: inflation in RR estimates

**Figure 4:**
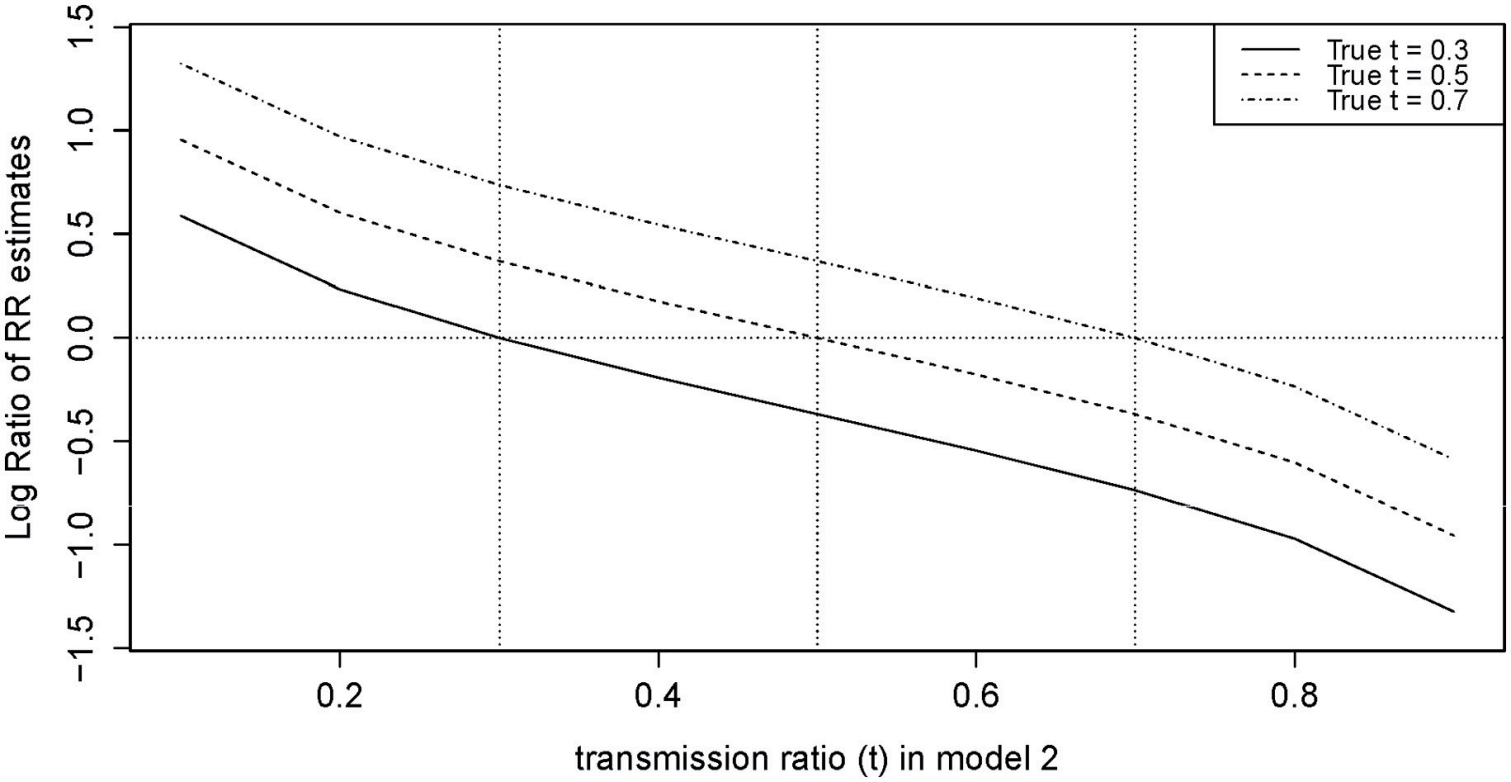
**Log ratio of RR in model 2 (Adjusted) for selected *t* (from 0.1 to 0.9) vs. True *t***.

We observed that using an under-estimated *t*-value in model 2 led to inflation, while an over-estimated *t* led to attenuation for *R*_**1**_ (Figure 4). We also noted that the inflation and attenuation of the log RR ratio was linear, which means exponential in arithmetic scale. When the difference between the true and selected *t* was ±0.1, the inflation ratio lied between 10^0.25^ = 1.78 and 10^−0.25^ = 0.56 for *R*_**1**_. When the difference was greater than ± 0.1, the inflation ratio became more pronounced. The slope of the log RR ratio curve for *R*_**2**_ was twice (not shown) that of *R*_**1**_ in Figure 4. Therefore, the inflation or attenuation in *R*_**2**_ was more severe than in *R*_**1**_. Results from our model 2 were highly sensitive to an incorrect input of *t*-value.

#### Sensitivity analysis: attenuation and inflation in power

**Figure 5:**
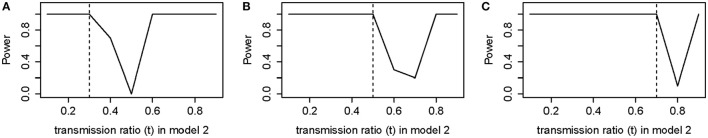
**Power of model 2 (Adjusted) for selected *t* (from 0.1 to 0.9) vs. true *t* (A) true *t* = 0.3 (B) true *t* = 0.5 (C) true *t* = 0.7**.

In Figures 5A,B, for *t* = 0.3 and 0.5, the power to detect true association was completely restored when the selected *t* was equal to the true *t*. However, setting the selected and true at *t* = 0.7 (Figure 5C), the power for detecting true association was not completely restored, consistent with what we observed previously in power analysis. There was a decrease in power when true signal is partially canceled by the selected *t.* We see that power was also highly sensitive to incorrect *t.*

### Application to a case-control, case-, and control-parent trio study of IUGR

The MAF calculated from all complete trios in our sample was 23.8% for *F13A1*, 46.4% for *PAI-1*, 27.1% for *MTHFR A1298C*, 28.9% for *MTHFR C677T*, 2.92% for *F5*, and 1.68% for *F2* (Tables 3, 4). Except for *MTHFR A1298C*, all MAF were close to the expected range from the literature (Kawamura et al., 1989; Ulvik et al., 1998; Ariens et al., 2002; Sapru et al., 2009; Alfirevic et al., 2010; Kvasnicka et al., 2012). Discrepancies were likely due to the fact that the samples were genetically heterogeneous with ~25% being black.

**Table 4:** **Relative risk with 95% CI, *P*-values, and LRT *P*-values of models 1 (Unadjusted) and 2 (Adjusted) for 4 thrombopilic genes (*F13A1, PAI-1, MTHFR A1298C*, and *MTHFR C677T*), With MAF and transmission ratio (*t*), on an intrauterine growth restriction dataset collected from a Canadian hospital between 1998 and 2000**.

**Gene**	**Model**	**MAF**	***t***	***R_1_***	**95%CI**	***R_1_ P*-value**	***R_2_***	**95%CI**	***R_2_ P*-value**	**LRT *P*-value**
*F13A1*	1	0.24	0.54	0.97	0.66, 1.43	0.89	1.41	0.68, 2.94	0.354	0.57
	2			0.82	0.56, 1.21	0.32	1.01	0.48, 2.1	0.98	0.55
*PAI-1*	1	0.46	0.49	0.80	0.49, 1.30	0.37	0.97	0.52, 1.82	0.93	0.53
	2			0.83	0.51, 1.35	0.46	1.06	0.57, 1.98	0.86	0.53
*MTHFR A1298C*	1	0.27	0.45	0.84	0.60, 1.19	0.34	0.78	0.40, 1.52	0.46	0.58
	2			1.04	0.74, 1.47	0.82	1.18	0.60, 2.31	0.63	0.89
*MTHFR C677T*	1	0.29	0.50	0.95	0.67, 1.35	0.8	0.75	0.39, 1.43	0.38	0.67
	2			0.94	0.67, 1.34	0.75	0.73	0.38, 1.40	0.34	0.65

R_1_, RR of cases carrying one copy of disease allele; R_2_, RR of cases carrying two copies of disease allele.

#### Application to 6 IUGR genes

We see in Table 4 that *F13A1, PAI-1*, and *MTHFR C677T* all had transmission ratios around 0.5. *MTHFR A1298C* had slightly lower transmission of the disease allele with *t* = 0.45. However, *F5* and *F2* had transmission deviate significantly from the Mendelian ratio with *t* = 0.36 and 0.11 (Table 5). RR from the loglinear model showed noassociation for *F13A1, PAI-1, MTHFR A1298C*, and *MTHFR C677T* variants (Table 4), similar to previous reports (Infante-Rivard et al., 2002, 2005). Due to the small number of genotype 2 cases for *F5* and *F2*, these two genes were analyzed under a dominant model. We see that for *F5*, conclusion on RR, *p*-values and LRT *p*-values are reversed from model 1 to model 2, suggesting a deleterious effect of the minor allele. For *F2*, we observed the opposite trend. The change in risk after adjustment for TRD was coherent with the expected effects from these variants given that they are known to affect placental circulation and thus potentially fetal growth.

**Table 5:** **Relative risk With 95% CI, *P*-values, LRT *P*-values of models 1 (Unadjusted) and 2 (Adjusted) for 2 thrombopilic genes (*F5* and *F2*), with MAF, transmission ratio (*t*) and Number of Genotype 2 Cases (G2), on an intrauterine growth restriction dataset collected from a Canadian Hospital Between 1998 and 2000**.

**Gene**	**Model**	**MAF**	***t***	**G2**	***R_1∕2_***	**95%CI**	***P*-value**	**LRT *P*-value**
*F5*	1	0.03	0.36	2	1.29	0.57, 2.93	0.54	0.53
	2				2.35	1.04, 5.33	0.04	0.039
*F2*	1	0.02	0.11	0	0.31	0.11, 0.85	0.023	0.014
	2				2.5	0.91, 6.82	0.074	0.1

R_1/2_, RR of cases carrying one or two copies of disease allele; Data is fitted with a dominant genotype model.

#### Comparison with TRD analysis in infante-rivard and Weinberg (2005) on FV gene

Infante-Rivard and Weinberg (2005) found in their study that both *F5* and *F2* exhibited evidence of TRD, as well as *MTHFR A1298C* but to a lesser extent, which is consistent with our estimation from control-trios (Tables 4, 5). The authors used six more strata from control-trios together with an interaction term between child genotype and case status. A gene-dosage model (*R*_**2**_ = *R*_**1**_^2^) was used implicitly to adjust for TRD; the RR for cases was estimated to be 3.59. We fitted model 2 using a gene-dosage model, and obtained a RR estimate of 2.88 with 95% CI: 1.31, 6.35. This result is in the range of the estimate from Infante-Rivard and Weinberg (2005). The number of trios included in these two analyses was different as Infante-Rivard and Weinberg (2005) used the LEM software with built-in EM algorithm for missing data whereas we only used complete trios. This shows that results from our extended loglinear model 2, which adjusts for TRD were comparable to those from the augmented model proposed in Infante-Rivard and Weinberg (2005).

The method proposed by Infante-Rivard and Weinberg (2005) requires fitting the loglinear model with actual control-trios, which is not required in our method where the transmission ratio of the minor allele is obtained through publicly available datasets. Therefore, less recruitment effort is needed leading to lower study cost. This difference is more significant for genome-wide studies where large samples are required.

Both models can include the same covariates. However, since control-trios are directly fitted in the model proposed by Infante-Rivard and Weinberg (2005), each covariate included in the model will lead to 2⋅of freedom loss because an interaction between case status (0, control; 1, case) and the covariate itself also has to be added. This leads to a faster decline in degrees of freedom than our method. The difference will further be magnified when other more complicated covariates, such as the mother-fetal interaction effect, are included in the model. Each of the four mother-fetal interaction covariates requires an additional interaction term with the case status.

The loglinear model proposed by Infante-Rivard and Weinberg (2005) allows missing data while our method requires complete trios only. The former has the advantage of using trios with missing parental genotypes, and hence does not need to discard trios with incomplete information. Currently, there is no immediate plan to augment our R-package for missing data, but it is possible in the future to address this issue using EM algorithm and include it as an option in our R-package. The loglinear model with control-trios has the advantage of adjusting for TRD without knowing the extent of distortion, and hence, remains a gold standard when the transmission ratio of the minor allele is not available.

## Discussion

Studies using animal models can potentially provide new insights in handling the phenomenon of TRD. TRD is much less studied in humans. In most genetic association studies in the current literature TRD remains largely unaccounted for. We previously reviewed a number of human studies on TRD (Naumova et al., 2001; Pardo-Manuel de Villena and Sapienza, 2001; Zöllner et al., 2004; Hanchard et al., 2005; The International HapMap Consortium, 2005; Paterson et al., 2009) and discussed the various methods and study designs in detecting TRD (Huang et al., 2013).

Here, we extend a model used for family-based association studies, accounting for TRD. Our simulation study showed that when TRD is unaccounted for as in model 1, the RR is inflated or attenuated exponentially. Power and Type 1 error also suffered greatly. Using a real dataset where the *F5* gene was studied as a determinant of IUGR, we validated our model in comparison with an approach using control trios (Infante-Rivard and Weinberg, 2005). However, we noted that the accuracy of our results depended on the correct TRD offset used in model 2. If we conduct a study with less well-known DSL and diseases, it is unlikely that we will have information on the TRD factor. Nevertheless, by leveraging on studies such as the HapMap project (The International HapMap Consortium, 2005), the 1000 Genomes Project (Auton et al., 2005), or the Framingham Heart Study (Framingham Heart Study, 2008), it may be possible to obtain such information.

The LEM software developed by van Den Oord and Vermunt (2000) that was used by Infante-Rivard and Weinberg (2005) to fit a loglinear model that takes into account of missing data. We compared RR estimates obtained from LEM and our models in the absence of TRD, and they were similar in values. HAPLIN, a software developed by Gjessing and Lie also studies case-parent-trios, which estimates the effect of multi-allelic markers or haplotype for single- and double-dose maternal and fetal haplotype (Gjessing and Lie, 2006). There are other software developed for studying case-parent trios such as TRANSMIT (Clayton and Jones, 1999), which can handle multi-locus haplotypes and missing parental information, and GASSOC (Schaid, 1996), which accommodate multi-allelic markers. These software do not readily have a component to adjust for TRD. However, we implemented the model 2 with TRD offset in an R package (named TRD) available on the Comprehensive R Archive Network (CRAN).

Currently, there is no comprehensive knowledge on TRD in the human genome. As TRD can inflate or attenuate an association signal, with large sets of SNPs being tested, results can be severely biased leading to spurious conclusions. Since TRD over generations leads to reduced mutational diversity in the genome, many of these TRD loci contain rare variants which are currently intensively researched. When transmission counts are small, even a slight distortion could lead to major impact on the outcome of the studies. Given what we observed in our simulation study, sequencing a control population to identify and quantify the extent of TRD in the human genome would seem necessary. Incorporating this information in the analysis of genetic association studies provides more accurate and valid estimates. Therefore, we suggest that knowledge of TRD in genomic databases is essential to determine the relevance of genes in various diseases.

## Author contributions

The research question for this manuscript was conceived by LH. AL reviewed and approved the conceived research question. CI acquired and provided the data used in this manuscript. LH developed, implemented and applied the method for simulation studies and real data analysis, and wrote the R software package “TRD.” AL contributed to a revision of the statistical model. LH drafted the manuscript. AL and CI reviewed it critically for important intellectual content. LH, AL, and CI all approved the final version to be published. LH, AL, and CI all agreed to be accountable for all aspects of work in ensuring the questions related to the accuracy or integrity of any part of the work are appropriately investigated and resolved.

## Funding

This work was supported in part by Dr. Aurélie Labbe from Canadian Institutes of Health Research Operating Grant MOP-93723.

### Conflict of interest statement

The authors declare that the research was conducted in the absence of any commercial or financial relationships that could be construed as a potential conflict of interest.
