# Supplementary material for: Analysis of Case-Parent Trios Using a Loglinear Model with Adjustment for Transmission Ratio Distortion
Source: Front Genet. 2016 Aug 31;7:155. doi: 10.3389/fgene.2016.00155 (PMC5005337; doi:10.3389/fgene.2016.00155)
Supplement: Supplementary file 1 [file Table6.docx]

**5. Appendix (Mathematical derivation)**

**5.1 Derivation of model 1 (without TRD offset) and 2 (with TRD offset)**

**5.1.1 Derivation of the general model**

Let M, F, and C represent the mother, father and child genotypes respectively. The 15 MFC genotype categories are described in Table 1. We also let *n_MFC_* represent the number of trios with genotypes MFC, and let D represent the disease status of the child. The probability of each MFC cell in Table 1 can be written as:

$P\left[ MFC | D \right]= E\left[ \frac{n_{MFC}}{n}|D \right]=\frac{P[D|MFC]P[C|MF]P\left[ MF \right]}{P[D]}$ (A1)

where

*P[D|MFC]* = Probability that the child is affected given a trio genotype MFC

*P[C|MF]* = Probability that the child genotype is C given parental genotypes MF

*P[MF]* = Probability of mating type MF for the parents

*P[D]* = Disease prevalence

Since we assume that there is no maternal or imprinting effect on the disease status of the child, we can write *P[D|MFC] = P[D|C]*, which means that the disease status of the child depends solely on the child's genotype. Furthermore, we re-write:

$P\left[ D | C \right]=P\left[ D | C=0 \right]\frac{P\left[ D | C \right]}{P\left[ D | C=0 \right]}=f_{0}R_{c}$ (A2)

where f_0_ is the penetrance factor for child genotype 0 and R_c_ is the RR of child genotype C, and C can be 1 or 2.

Therefore, equation (A1) can be written as:

$log\left\{ E \left[ \frac{n_{MFC}}{n}|D \right] \right\}=log P\left[ D | C \right]+log P\left[ C | MF \right]+log P\left[ MF \right]-log P[D]$

Using the notations $P\left[ C | MF \right]=\tau_{MFC}$, $P\left[ MF \right]=\mu_{MF}$, and $P\left[ D \right]=d$ (see Table 1), and using equation (A2) for *P[D|C],* we obtain:

$log\left\{ E \left[ n_{MFC}|D \right] \right\}=log\left( f_{0}R_{c} \right)+log \tau_{MFC}+ log \mu_{MF}+log n-log d$

$=log\left( \frac{f_{0}n}{d} \right)+log \tau_{MFC}+ log \mu_{MF}+\beta_{c}$ (A3)

where $log(R_{c})$ = $\beta_{c}$.

Model 1 described in this paper corresponds to the scenario where t = 0.5 is substituted into $\tau_{MFC}$ (Mendelian transmission). Model 2 corresponds to the scenario where t is not restricted to 0.5, and can take on values between 0 and 1, excluding 0 and 1.

**5.1.2 Statistical equation for model 1**

In order to fit the model described in equation (A3), we use different grouping schemes for model 1 and model 2. For Weinberg's model (model 1), the terms ${log(\tau}_{MFC})$ and $log (\mu_{MF})$ are grouped together, which we temporarily term $\varphi_{MF}$ plus an offset term, $log(2)I_{\left[ MFC=111 \right]}$, which only appears for MFC category 111 (seen in last column of Table 6). This is because it is the same within each stratum, except for stratum 4, where the sum ${log(\tau}_{MFC})+log(\mu_{MF})$ in Table 6 (last column) for MFC=111 is 2 times of MFC=112 and 110.

Therefore, to derive the statistical equation for model 1, equation (A3) can be re-written as

$log\left\{ E \left[ n_{MFC}|D \right] \right\}=log\left( \frac{f_{0}n}{d} \right)+\sum_{MF=mf} \varphi_{MF} I_{\left[ MF=mf \right]}+log\left( 2 \right)I_{\left[ MFC=111 \right]}+\beta_{1}I_{\left[ C=1 \right]}+\beta_{2}I_{\left[ C=2 \right]}$

We can then absorb the constant term $\frac{f_{0}n}{d}$ into the summation of $\varphi_{MF}$ terms and have

$log\left\{ E \left[ n_{MFC}|D \right] \right\}=\sum_{MF=mf} log{\left[ \left( \frac{f_{0}n}{d} \right)exp(\varphi_{MF}) \right]I}_{\left[ MF=mf \right]}+log\left( 2 \right)I_{\left[ MFC=111 \right]}+\beta_{1}I_{\left[ C=1 \right]}+\beta_{2}I_{\left[ C=2 \right]}$

By noting $\gamma_{MF}$ as the first term of the above equation, model 1 can be written as:

$log\left\{ E \left[ n_{MFC}|D \right] \right\}=\sum_{MF=mf} {\gamma_{MF}I}_{\left[ MF=mf \right]}+log\left( 2 \right)I_{\left[ MFC=111 \right]}+\beta_{1}I_{\left[ C=1 \right]}+\beta_{2}I_{\left[ C=2 \right]}$

Since there are 6 strata (S) of MF mating types, by fitting the model with an intercept, we finally obtain:

$log\left\{ E \left[ n_{MFC}|D \right] \right\}=\rho_{6}+\sum_{j=1}^{5} \rho_{j}I_{\left[ S=j \right]}+log\left( 2 \right)I_{\left[ MFC=111 \right]}+\beta_{1}I_{\left[ C=1 \right]}+\beta_{2}I_{\left[ C=2 \right]}$ (A4)

where $\gamma_{6}=\rho_{6}$ and $\gamma_{j}=\rho_{6}+\rho_{j}$ for j = 1 to 5.

**5.1.3 Statistical equation for model 2**

For model 2, we do not group the terms ${log(\tau}_{MFC})$ and $log (\mu_{MF})$ together, but assign ${log(\tau}_{MFC})$ as an offset given a specific value of t (Table 1), and estimate $log (\mu_{MF})$. Therefore, equation (A3) can be re-written as:

$log\left\{ E \left[ n_{MFC}|D \right] \right\}=log\left( \frac{f_{0}n}{d} \right)+ \sum_{MF=mf} log \mu_{MF}I_{[MF=mf]}+log \tau_{MFC}+\beta_{1}I_{\left[ C=1 \right]}+\beta_{2}I_{\left[ C=2 \right]}$

$=\sum_{MF=mf} {log\left( \frac{f_{0}n}{d} \right)\mu}_{MF}I_{[MF=mf]}+log \tau_{MFC}+\beta_{1}I_{\left[ C=1 \right]}+\beta_{2}I_{\left[ C=2 \right]}$

By noting ${log\left( \frac{f_{0}n}{d} \right)\mu}_{MF}$ as $\alpha_{MF}$, model 2 can be written as:

$log\left\{ E \left[ n_{MFC}|D \right] \right\}=\sum_{MF=mf} \alpha_{MF}I_{[MF=mf]}+log \tau_{MFC}+\beta_{1}I_{\left[ C=1 \right]}+\beta_{2}I_{\left[ C=2 \right]}$

By fitting the model with an intercept, we finally obtain:

$log\left\{ E \left[ n_{MFC}|D \right] \right\}=\xi_{6} {+\sum_{j=1}^{5} \xi_{j}I}_{[S=j]}+log \tau_{MFC}+\beta_{1}I_{\left[ C=1 \right]}+\beta_{2}I_{\left[ C=2 \right]}$ (A5)

where $\alpha_{6}=\xi_{6}$ and $\alpha_{j}=\xi_{6}+\xi_{j}$ for j = 1 to 5 and *S* = stratum.

Therefore, final statistical formula for model 1 is written in equation (A4) and for model 2 in equation (A5).

**5.2: Non-Central Chi-square Likelihood for model 1 (without TRD offset) and model 2 (with TRD offset)**

To perform the Likelihood Ratio Test (LRT) in assessing significance of association between the disease phenotype and DSL, we set up a null model for both model 1 and 2 with null hypothesis H_0_ : $\beta_{1}=\beta_{2}=0$. The corresponding LRT test statistic, which is the difference in deviance between null and full model, has an asymptotic Chi-Square distribution with 2 degrees of freedom accounting for the two extra terms R_1_ and R_2_. Agresti (1) showed that when the null hypothesis is not true for a loglinear model, the resulting LRT is a chi-square statistic with a non-centrality parameter (NCP):

$\lambda=2n\sum_{MFC} \pi_{MFC}(M_{a})log \left( \frac{\pi_{MFC}(M_{a})}{\pi_{MFC}(M_{0})} \right)$

where $\pi_{MFC}(M_{a})$ is the true probability of each cell with MFC combination, and $\pi_{MFC}(M_{0})$ is the value under the null hypothesis. We also denoted the degree of freedom as $\upsilon$, which is 2 in our LRT because there are 2 extra variables R_1_ and R_2_ in the alternative model than the corresponding null model.

To calculate Type 1 error and power comparable to our theoretical values, we need to have the exact likelihood. Our likelihood for the alternative hypothesis is shown in equation (A1) and re-written as:

$\pi_{MFC}\left( M_{a} \right)=\frac{f_{0}R_{c}\tau_{MFC}\mu_{MF}}{d}$

where $f_{0}R_{c}$, $\tau_{MFC}$, $\mu_{MF}$ and $d$ are defined as in equation (A2) and (A3).

In the presence of TRD, we know that even when the null hypothesis is true, the LRT still has a non-Central Chi-square distribution. The null model is different for models 1 and 2 because TRD is being adjusted in the offset of model 2 but not in model 1. Under the null hypothesis, *P[D|MFC] = P[D]*, and hence, *f_0_R_c_/d* = 1. The likelihoods for models 1 and 2 under null hypothesis are then, respectively:

$\pi_{MFC}\left( M_{01} \right)=\mu_{MF} \tau_{MFC} [0.5]$

and

$\pi_{MFC}\left( M_{02} \right)=\mu_{MF} \tau_{MFC}[t]$

Under the alternative hypothesis, NCP for model 1 is:

$\lambda_{1}=2n\sum_{MFC} \frac{f_{0}{R_{c}\tau_{MFC}\mu}_{MF}[t]}{d} log\left( \frac{f_{0}R_{c}\tau_{MFC}[t]}{\tau_{MFC}[0.5] d} \right)$ (A6)

and the NCP for model 2 is:

$\lambda_{2}=2n\sum_{MFC} \frac{f_{0}{R_{c}\tau_{MFC}\mu}_{MF}[t]}{d} log\left( \frac{f_{0}R_{c}}{d} \right)$ (A7)

Note that when t is not equal to 0.5, even though there is no association signal, the LRT is still a NCP chi-square statistic. The NCP for model 1 is 0 when t = 0.5 (Mendelian transmission) and $\frac{f_{0}R_{c}}{d}$=1 (no association). Therefore, null hypothesis for model 1 requires both Mendelian transmission and no association between disease and DSL. However, since TRD has already been adjusted for in model 2, the NCP is 0 when $\frac{f_{0}R_{c}}{d}$=1 (no association).

Table 6: Stratum Frequency and Probability of Transmission (Mendelian) for Case-parent Trios Study Design

| Stratum | MF genotype | C genotype | Stratum frequency ($\mu_{MF}$) | Probability of transmission $(\tau_{MFC})$ | ${log(\mu}_{MF})+log\left( \tau_{MFC} \right)=\varphi_{MFC}+log(2)I_{\left[ MFC=111 \right]}$ |
| --- | --- | --- | --- | --- | --- |
| 1 | 22 | 2 | *p^4^* | 1 | *log[p^4^]+0* |
| 2 | 21 or 12 | 1 or 2 | *2p^3^(1-p)* | 1/2 | *log[p^3^(1-p)]+0* |
| 3 | 20 or 02 | 1 | *p^2^(1-p)^2^* | 1 | *log[p^2^(1-p)^2^]+0* |
| 4 | 11 | 2 | *4p^2^(1-p)^2^* | 1/4 | *log[p^2^(1-p)^2^]+0* |
|  | 11 | 1 | *4p^2^(1-p)^2^* | 1/2 | *log[p^2^(1-p)^2^]+log2* |
|  | 11 | 0 | *4p^2^(1-p)^2^* | 1/4 | *log[p^2^(1-p)^2^]+0* |
| 5 | 10 or 01 | 0 or 1 | *2p(1-p)^3^* | 1/2 | *log[p(1-p)^3^]+0* |
| 6 | 00 | 0 | *(1-p)^4^* | 1 | *log[(1-p)^4^]+0* |

**REFERENCES**

1. Agresti A. Building and Applying Logistic Regression Models. Cateigorical Data Analysis 2nd Ed. Hoboken, NJ, USA: John Wiley & Sons, Inc.; 2002. p. 243-4.
